# Gut microbiota causally affects drug-induced liver injury via plasma metabolites: a Mendelian randomization study

**DOI:** 10.3389/fmicb.2024.1432049

**Published:** 2024-07-18

**Authors:** Haoshuang Fu, Shuang Zhao, Shuying Song, Qing Xie

**Affiliations:** ^1^Department of Infectious Diseases, Ruijin Hospital, Shanghai Jiao Tong University School of Medicine, Shanghai, China; ^2^Department of Critical Liver Diseases, Liver Research Center, Beijing Friendship Hospital, Capital Medical University, National Clinical Research Center for Digestive Diseases, Beijing, China

**Keywords:** toxic liver disease, drug-induced liver disease, gut microbiota, plasma metabolome, Mendelian randomization, liver disease

## Abstract

**Background:**

The gut microbiota and plasma metabolites play important roles in the progression of drug-induced liver injury (DILI). We investigated the causal associations between the gut microbiota, plasma metabolome, and DILI.

**Methods:**

The summary data for gut microbiota (*n* = 18,340), plasma metabolome (*n* = 8,299), and DILI (*n* = 366,838) were obtained from the large genome-wide association studies. A two-sample Mendelian randomization was performed to explore the associations between the gut microbiota, plasma metabolome, and DILI. Additionally, a two-step Mendelian randomization was performed to explore the potential metabolites.

**Results:**

Five taxa were causally associated with DILI, including *Oscillospira* [odds ratio (OR) = 2.257, 95% confidence interval (CI) = 1.110–4.590], *Blautia* (OR = 2.311, 95% CI = 1.010–5.288), *Roseburia* (OR = 2.869, 95% CI = 1.429–5.761), *Fusicatenibacter* (OR = 1.995, 95% CI = 1.024–3.890), and *Prevotella 7* (OR = 1.549, 95% CI = 1.065–2.253). Moreover, 53 metabolites were causally associated with DILI. After mediation analysis, four taxa were found to affect DILI through five mediation metabolites. N6-carbamoylthreonyladenosine mediated the effect of *Blautia* on DILI. Acetylcarnitine mediated the effect of *Fusicatenibacter* on DILI. In addition, 4-cholesten-3-one mediated the effect of *Prevotella 7* on DILI. Furthermore, 5,6-dihydrothymine levels and the salicylate-to-citrate ratio mediated the effect of *Oscillospira* on DILI.

**Conclusion:**

We found that the gut microbiota could affect DILI through plasma metabolites, which could serve as potential biomarkers for risk stratification and elucidate underlying mechanisms for further investigation of DILI.

## Introduction

Drug-induced liver injury (DILI) is liver damage caused by various chemicals and drugs (European Association for the Study of the Liver et al., 2019), affecting 13%−15% of patients with acute liver failure in Western countries (European Association for the Study of the Liver et al., 2019). Compared to other etiologies, patients with DILI present worse transplant-free survival (European Association for the Study of the Liver et al., 2019). Therefore, it is imperative to investigate the occurrence and progression of DILI.

The gut microbiota has been reported to play a pivotal role in liver disease (Chu et al., 2023). However, epidemiological evidence on the associations between the gut microbiota and DILI is conflicting. For example, it has been reported that the abundance of *Blautia* decreased in mice with DILI compared to controls (Zhang et al., 2022), whereas the abundance of *Blautia* was reported to increase in mice with DILI compared to controls in other studies (Wang et al., 2022). Conflicting evidence on the association between *Bacteroides* and DILI was also reported (Chu et al., 2023). The causal effects of the gut microbiota on DILI and its underlying mechanisms remain unclear.

Interestingly, it has been found that the gut microbiota could affect DILI by modulating blood metabolites such as adrenic acid and aspartic acid (Zhao et al., 2022). Given the potential associations between the gut microbiota, blood metabolites, and DILI, we investigated the causal associations between the gut microbiota, plasma metabolome, and DILI, aiming to provide potential targets for risk stratification and to elucidate the underlying mechanisms for further investigation of DILI.

Mendelian randomization, an instrumental variable method utilizing single nucleotide polymorphisms (SNPs) as instruments, has been employed to elucidate the causal associations between two features. Mendelian randomization offers two advantages: mitigating bias resulting from confounding factors and addressing issues related to reverse causality (Emdin et al., 2017). Therefore, we performed a Mendelian randomization analysis to investigate the causal associations between the gut microbiota, plasma metabolome, and DILI.

## Methods

### Study design

**Figure 1:**
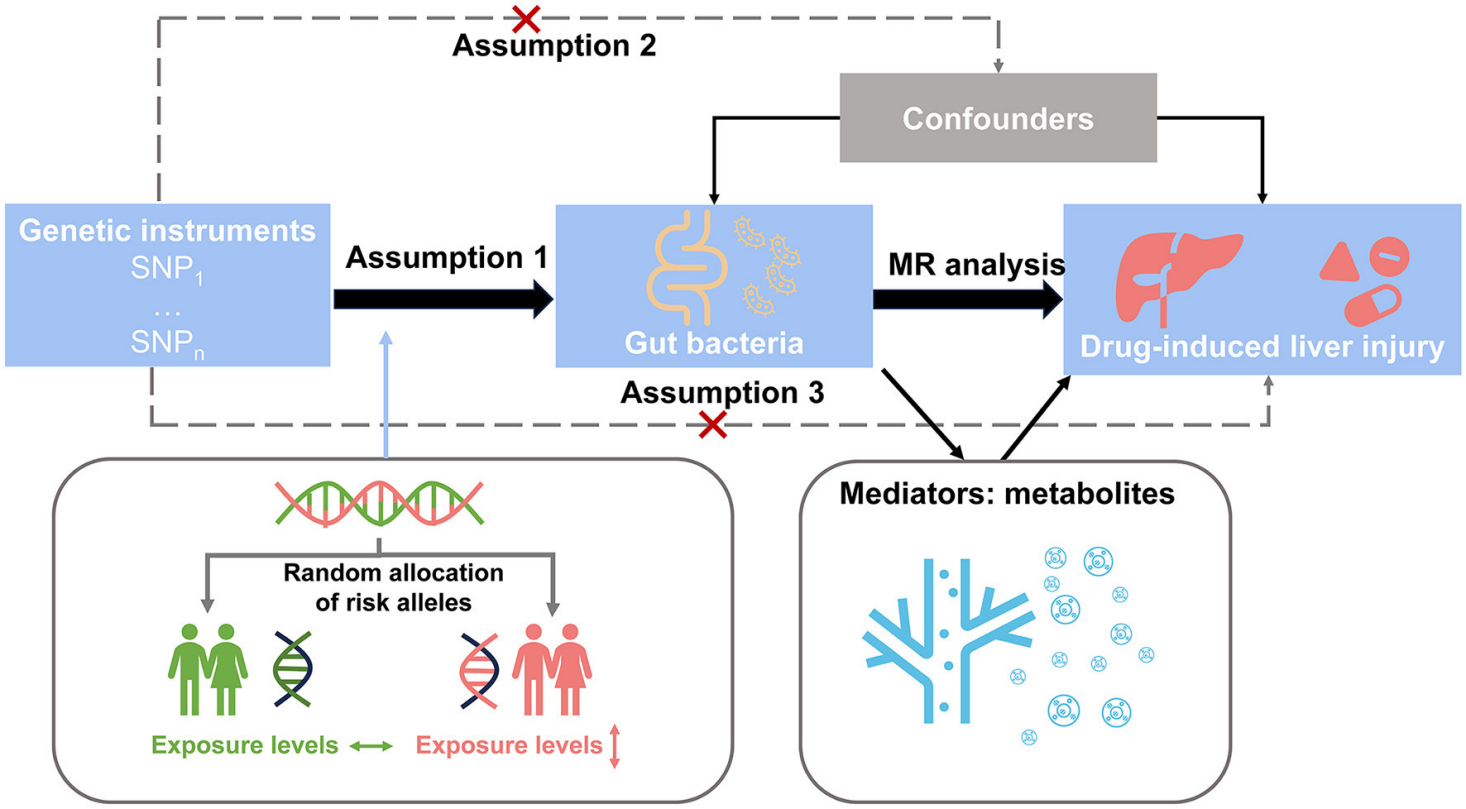
Study design overview. Step 1: We identified SNPs as instrument variables and performed univariable Mendelian randomization analysis to explore the causal effects of the gut microbiota and plasma metabolome on drug-induced liver injury. Step 2: We implemented two-step Mendelian randomization to identify potential metabolites as mediators in the associations between the gut microbiota and drug-induced liver injury.

The study design is shown in Figure 1. Initially, we identified SNPs as instrument variables and performed a univariable Mendelian randomization analysis to explore the causal effects of the gut microbiota and plasma metabolomes on DILI. Next, we implemented a two-step Mendelian randomization to identify potential metabolites as mediators in the associations between the gut microbiota and DILI. Our study was performed in accordance with the Strengthening the Reporting of Observational Studies in Epidemiology Reporting Guidelines (Skrivankova et al., 2021) (Supplementary material).

### Data sources

Genome-wide association study (GWAS) data sources are summarized in Table 1. All procedures followed were in accordance with the ethical standards of the responsible committee on human experimentation (institutional and national) and with the Declaration of Helsinki of 1975, as revised in 2008. All studies included in the cited genome-wide association studies had been approved by a relevant review board. All participants provided informed consent.

**Table 1:** Data sources.

**Trait**	**Participants**	**Ancestry**	**Dataset source**	**Year**	**PubMed ID**
Gut microbiota	18,340	Mixed (78% European)	MiBioGen	2021	33462485
Blood metabolites	8,299	European	GWAS Catalog	2023	36635386
Drug-induced liver injury	388 cases and 366,450 controls	European	FinnGen	2023	36653562

GWAS data on the gut microbiota were obtained from the MiBioGen consortium based on 18,340 participants, of which 78% were Europeans (Kurilshikov et al., 2021). The MiBioGen consortium analyzed the genome information and 16S fecal microbiome, which resulted in 211 taxa (131 genera, 35 families, 20 orders, 16 classes, and nine phyla). Only data from 131 genera were used in our study. Moreover, GWAS data on plasma metabolomes were obtained from 8,299 European individuals in the Canadian Longitudinal Study on Aging cohort, including 1,091 metabolites and 309 metabolite ratios (Chen et al., 2023). Furthermore, GWAS data for DILI were obtained from the FinnGen consortium, including 388 cases and 366,450 controls (Kurki et al., 2023), which were diagnosed based on the International Classification of Diseases (ICD10: K71). Refer to the cited papers for more detailed information on each GWAS data source. Overlapping individuals have negligible effects on power when selected instrument variables are strong enough (Pierce and Burgess, 2013).

### Selection of instrument variables

SNPs for the gut microbiota and plasma metabolites were selected as instrument variables with a *p*-value (< 1 × 10^−5^). All SNPs were clumped for independent inheritance (*R*^2^ < 0.001, within 10 Mb). *F*-statistics were calculated to assess SNPs' validity, with a threshold of exceeding 10 (Pierce and Burgess, 2013).

### Statistical analysis

A univariable Mendelian randomization analysis was performed to explore the causal effects of the gut microbiota and plasma metabolomes on DILI. Five methods [inverse-variance-weighted (IVW), MR-Egger, weighted median, simple mode, and weighted mode] were used to evaluate and validate causal effects, and assumptions and advantages are summarized in Supplementary Table S1. Inverse-variance-weighted was the primary method used. Cochran's *Q* statistic was calculated to evaluate heterogeneity. The MR-Egger intercept was performed to evaluate pleiotropy. Only a causal effect with inverse-variance-weighted *p*-value of < 0.05 and without heterogeneity and pleiotropy (corresponding *p*-value of >0.05) was identified as a significant effect. Moreover, based on the above criteria, we selected more stringent criteria with consistent directionality across the five methods to analyze the associations between plasma metabolomes and DILI. Furthermore, we used MetaboAnalyst 5.0 (https://www.metaboanalyst.ca/) to implement the enrichment analysis of identified metabolites. Furthermore, reverse causality between the gut microbiota and DILI was assessed using the above methods.

A two-step Mendelian randomization (Burgess et al., 2015) was used to explore the mediation roles of plasma metabolites in the causal effects of the gut microbiota on DILI. Based on the causal effects (β_EO_) of the gut microbiota on DILI, two causal effects were explored: the causal effects (β_MO_) of mediators (plasma metabolites) on DILI and the causal effects (β_EM_) of exposures (the gut microbiota) on mediators. β_EM_ × β_MO_ represented the mediation effect (β_mediation_). Where there was evidence that the gut microbiota affected plasma metabolites, which in turn affected DILI, we used the product of the coefficient method to evaluate mediation effects. The mediation proportion was calculated using the following formula: β_mediation_/β_EO._ Standard errors for mediation effects were derived using the delta method.

All tests were two-sided and performed using the TwoSampleMR (version 0.5.7) packages in R software (version 4.0.2). All IVW results were corrected for multiple testing using the false discovery rate (FDR) method. An FDR *p*-value of < 0.05 was identified as statistically significant. A *p*-value of < 0.05 was identified as nominally significant.

## Results

### Identification of genetic instruments

SNPs for each exposure and mediator were identified as genetic instruments based on the selection criteria. All SNP F-statistics exceeded 10, indicating enough validity (Supplementary Tables S2–S4).

### Causal effects of the gut microbiota on DILI

**Figure 2:**
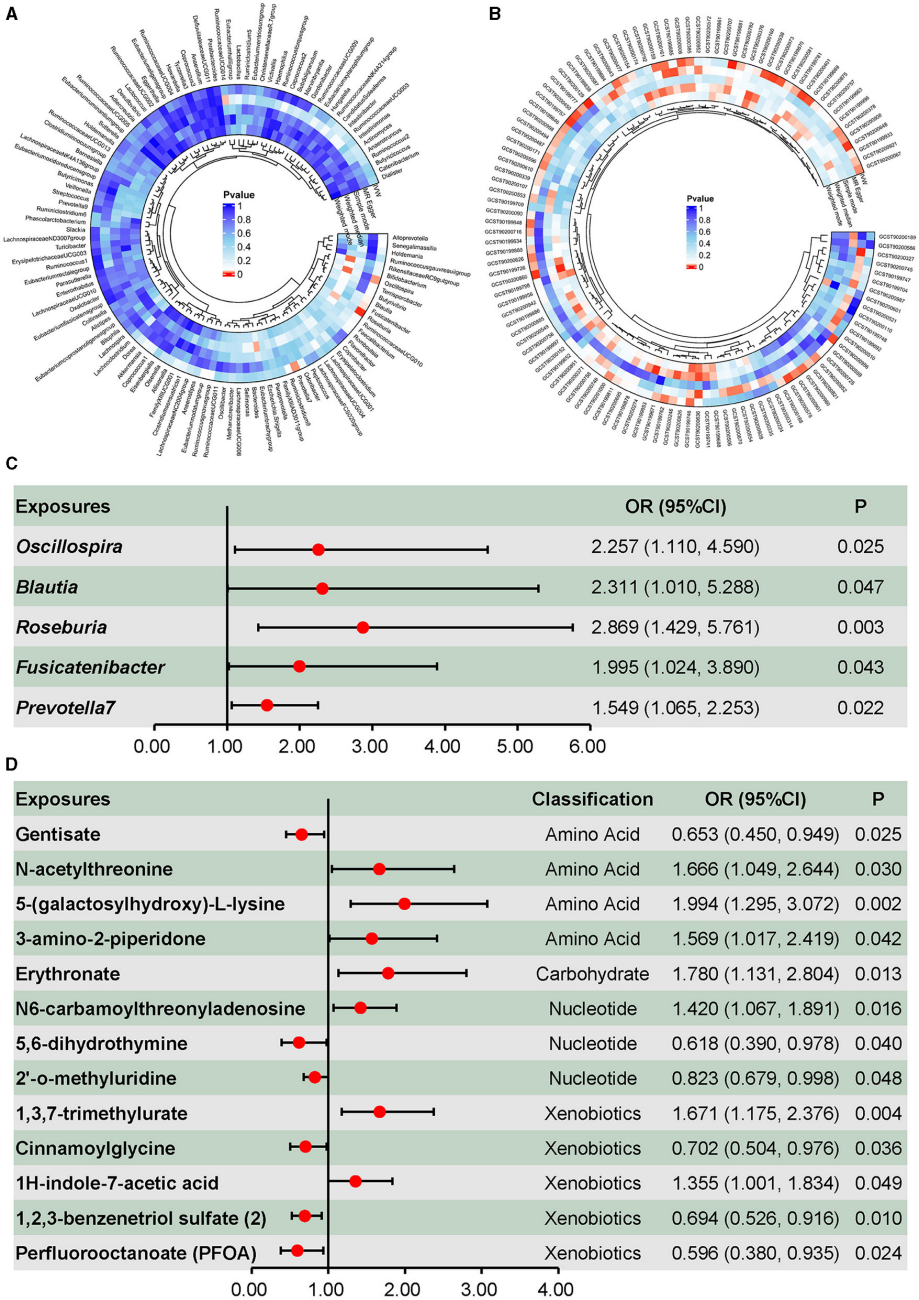
Causal effects of the gut microbiota and plasma metabolites on drug-induced liver injury. **(A)** Screening of the causal effects of the gut microbiota on drug-induced liver injury. **(B)** Screening of the causal effects of plasma metabolites on drug-induced liver injury. **(C)** Causal effects of the gut microbiota on drug-induced liver injury. **(D)** Causal effects of plasma amino acids, carbohydrate, nucleotides, and xenobiotics on drug-induced liver injury.

As shown in Figure 2A, the causal effects of the gut microbiota on DILI were explored, and five taxa presented causal effects on DILI using the inverse-variance-weighted method without heterogeneity or pleiotropy. As shown in Figure 2C, these taxa were all associated with an increasing risk of DILI. *Oscillospira* (OR = 2.257, 95% CI = 1.110–4.590, *p* = 0.025), *Blautia* (OR = 2.311, 95% CI = 1.010–5.288, *p* = 0.047), and *Roseburia* (OR = 2.869, 95% CI = 1.429–5.761, *p* = 0.003) presented causal effects on the increasing risk of DILI, as well as *Fusicatenibacter* (OR = 1.995, 95% CI = 1.024–3.890, *p* = 0.043) and *Prevotella 7* (OR = 1.549, 95% CI = 1.065–2.253, *p* = 0.022). Only nominally significant effects were detected rather than statistically significant effects. No reverse causality was detected in the above associations (Supplementary Table S5). Neither heterogeneity nor pleiotropy was detected in the above associations (Supplementary Table S6).

### Causal effects of plasma metabolites on DILI

**Figure 3:**
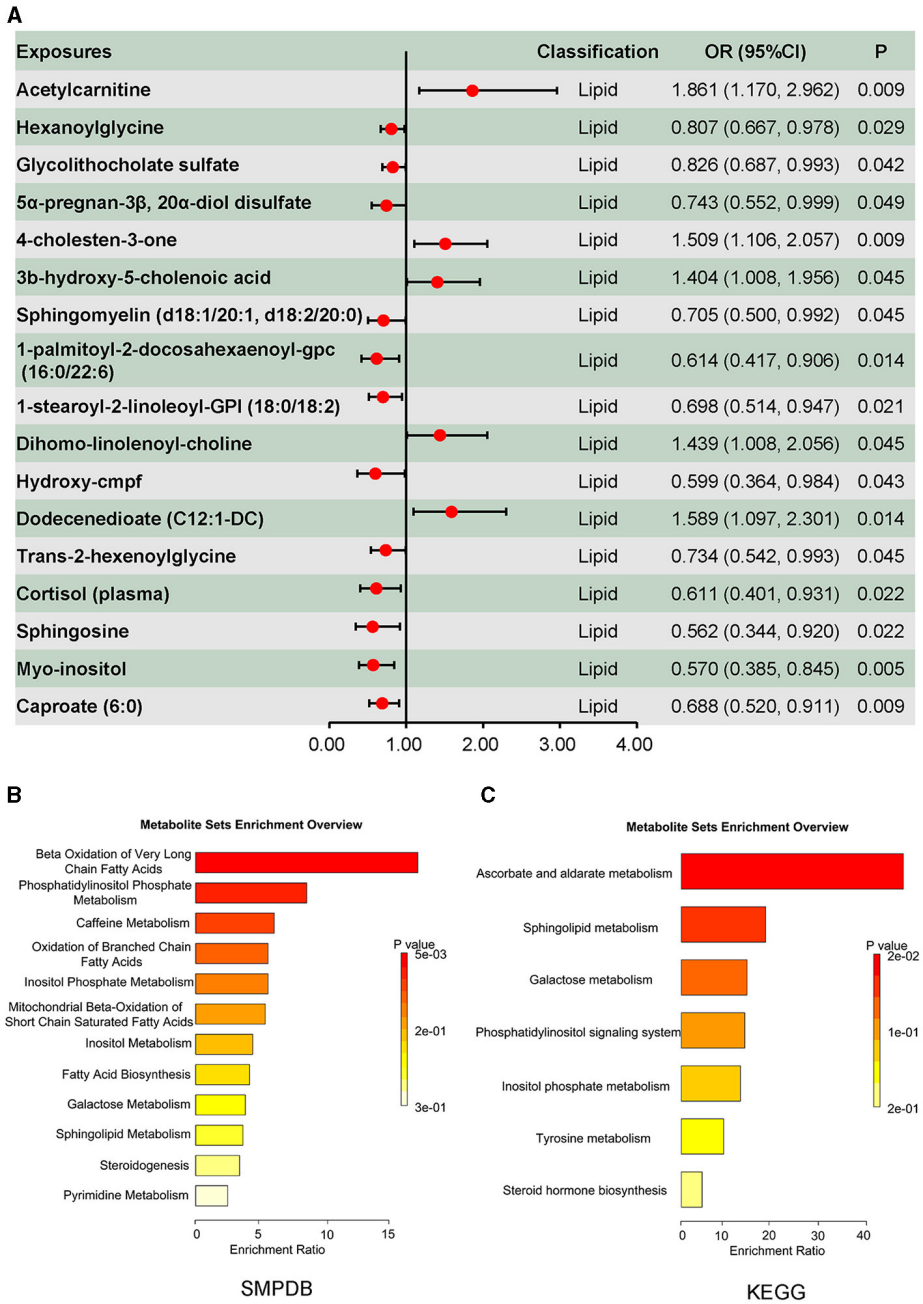
Causal effects of plasma lipid on drug-induced liver injury, and the enrichment analysis results of the causal effect of plasma metabolites on drug-induced liver injury. **(A)** Causal effects of plasma lipid on drug-induced liver injury. **(B)** Enrichment analysis results of the causal effect of plasma metabolites on drug-induced liver injury based on Small Molecule Pathway Database (SMPDB). **(C)** Enrichment analysis results of the causal effect of plasma metabolites on drug-induced liver injury based on the KEGG database.

Metabolites associated with DILI are summarized in Figure 2B, in which 53 metabolites were identified using the inverse-variance-weighted method and with persistent directionality across all five methods, without heterogeneity or pleiotropy. Among these 53 metabolites, there were 30 known metabolites, including amino acids, carbohydrates, nucleotides, xenobiotics, and lipids. Causal associations between DILI and four amino acids, one carbohydrate, three nucleotides, and five xenobiotics are shown in Figure 2D, including seven positive and six negative causal effects on DILI. Moreover, 17 lipids presented causal effects on DILI, including five positive and 12 negative effects (Figure 3A). Based on known metabolites, enrichment analysis indicated that they were enriched in the beta-oxidation pathways of very long-chain fatty acids and ascorbate and aldarate metabolism (Figures 3B, C).

Furthermore, 14 metabolite ratios and nine unknown metabolites were associated with the DILI (Table 2). Only nominally significant effects were detected rather than statistically significant effects. Neither heterogeneity nor pleiotropy was detected in the above associations (Supplementary Table S6).

**Table 2:** Causal effects of plasma metabolite ratios and unknown metabolites on drug-induced liver injury.

**Exposures**	**Classification**	**OR (95% CI)**	***p*-value**
X-12127	Unknown	0.705 (0.514, 0.967)	0.030
X-11880	Unknown	0.615 (0.387, 0.976)	0.039
X-17653	Unknown	0.724 (0.537, 0.975)	0.033
X-18901	Unknown	1.427 (1.002, 2.033)	0.049
X-18922	Unknown	0.681 (0.516, 0.899)	0.007
X-21441	Unknown	1.201 (1.005, 1.434)	0.043
X-23655	Unknown	0.633 (0.432, 0.927)	0.019
X-23593	Unknown	0.668 (0.453, 0.983)	0.041
X-24585	Unknown	0.584 (0.403, 0.845)	0.004
Adenosine 5′-diphosphate (ADP)-to-phosphoethanolamine ratio	Ratio	1.442 (1.035, 2.009)	0.031
Mannose-to-trans-4-hydroxyproline ratio	Ratio	0.649 (0.434, 0.970)	0.035
Spermidine-to-choline ratio	Ratio	1.937 (1.086, 3.454)	0.025
Adenosine 5′-diphosphate (ADP)-to-glycine ratio	Ratio	1.286 (1.018, 1.625)	0.035
Adenosine 5′-monophosphate (AMP)-to-serine ratio	Ratio	2.383 (1.315, 4.317)	0.004
Cysteinylglycine-to-glutamate ratio	Ratio	0.699 (0.492, 0.994)	0.046
Ornithine-to-phosphate ratio	Ratio	0.543 (0.325, 0.906)	0.019
Mannose-to-mannitol-to-sorbitol ratio	Ratio	1.606 (1.037, 2.489)	0.034
Alpha-ketoglutarate-to-alanine ratio	Ratio	0.603 (0.414, 0.878)	0.008
Glutamate-to-pyruvate ratio	Ratio	1.461 (1.003, 2.128)	0.048
Cytidine-to-N-acetylglucosamine-to-N-acetylgalactosamine ratio	Ratio	0.547 (0.354, 0.846)	0.007
Proline-to-glutamate ratio	Ratio	0.651 (0.482, 0.880)	0.005
Phosphoethanolamine-to-choline ratio	Ratio	0.570 (0.364, 0.891)	0.014
Salicylate-to-citrate ratio	Ratio	0.648 (0.437, 0.961)	0.031

### Mediation analysis of plasma metabolites

**Figure 4:**
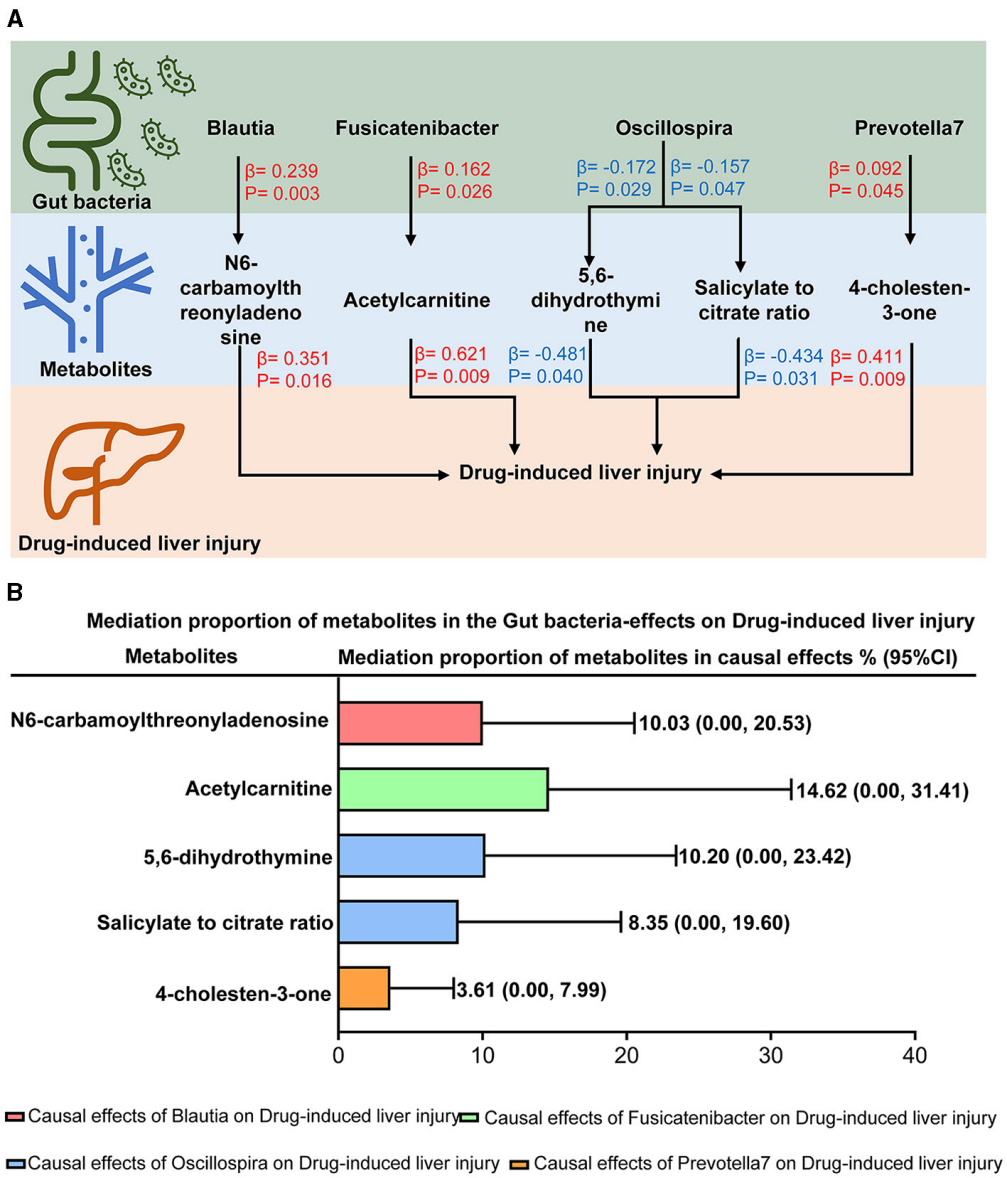
Mediation effects of plasma metabolites on the causal effects of the gut microbiota on drug-induced liver injury. **(A)** The mediation mode of plasma metabolites on the causal effects of the gut microbiota -on drug-induced liver injury in the mediation analysis. β indicated the causal effects using inverse-variance-weighted method (*p* < 0.05). Red-β and blue-β signified positive and negative effects, respectively. **(B)** Mediation proportion of plasma metabolites on the effects of the gut microbiota on drug-induced liver injury.

A two-step Mendelian randomization was implemented based on the identified gut taxa and plasma metabolites to explore the mediation roles of plasma metabolites in the effects of the gut microbiota on DILI. As shown in Figure 4A, five mediation pathways were identified. N6-carbamoylthreonyladenosine mediated 10.03% (95% CI = 0.00%−20.53%, Figure 4B) of the effect of *Blautia* on DILI. Acetylcarnitine mediated 14.62% (95% CI = 0.00%−31.41%, Figure 4B) of the effect of *Fusicatenibacter* on DILI. In addition, 4-cholesten-3-one mediated 3.61% (95% CI = 0.00%−7.99%, Figure 4B) of the effect of *Prevotella 7* on DILI. Moreover, 5,6-dihydrothymine levels and the salicylate-to-citrate ratio mediated 10.20% (95% CI = 0.00%−23.42%, Figure 4B) and 8.35% (95% CI = 0.00%−19.60%, Figure 4B) of the effect of *Oscillospira* on DILI, respectively. Detailed information for mediation analysis is summarized in Supplementary Table S7. These findings indicated that the gut microbiota could affect DILI via plasma metabolites.

## Discussion

In this study, we systemically investigated the causal effects of the gut microbiota and blood metabolites on DILI. We revealed that five taxa and 53 metabolites were causally associated with DILI. Further analysis showed that four taxa affected DILI via five mediation metabolites, which could serve as potential biomarkers for risk stratification and as underlying mechanisms for further investigation of DILI.

*Blautia* and *Fusicatenibacter* positively affected DILI by modulating plasma N6-carbamoylthreonyladenosine and acetylcarnitine levels, respectively. A previous study found that the abundance of the *Balutia* genus increased in mice with methotrexate-induced liver injury (Wang et al., 2022), whereas it decreased in mice with triclosan-induced liver injury (Zhang et al., 2022). Our result supported the hypothesis that *Blautia* was positively associated with DILI. Besides, we found that *Blautia* could positively affect DILI via modulating plasma N6-carbamoylthreonyladenosine levels. Similarly, we revealed that *Fusicatenibacter* exerted a positive causal effect on DILI by modulating plasma acetylcarnitine levels. *Fusicatenibacter* was reported to increase in patients with hepatic cirrhosis and portal vein thrombosis (Huang et al., 2023; Sato et al., 2023). Our result further investigated the role of *Fusicatenibacter* in DILI (Alotaibi et al., 2016). Interestingly, we found that *Fusicatenibacter* positively affects DILI through increasing acetylcarnitine levels, which is the underlying mechanism that needs to be explored in future studies.

*Prevotella 7* exerted a positive effect on DILI via modulating the plasma 4-cholesten-3-one level. Based on the urine bacteria analysis, the abundance of *Prevotella* was reported to be increased in patients with anti-tuberculosis drug-induced hepatotoxicity (Wu et al., 2022). Moreover, a previous study found that Prevotella was enriched in mice fed a Western diet, resulting in aggravated carbon tetrachloride-induced liver injury (Yang et al., 2021). Increasing plasma 4-cholesten-3-one levels were reported as a biomarker for non-alcoholic fatty liver disease risk (Pang et al., 2022). Our study indicated the new role of 4-cholesten-3-one due to increased *Prevotella 7* and the underlying mechanism of DILI.

*Oscillospira* positively affected DILI by modulating plasma 5,6-dihydro thymine levels and the salicylate-to-citrate ratio. Observational studies on the associations between *Oscillospira* and liver disease are conflicting. It was reported that *Oscillospira* increased in fructose-treated mice, activating the lipopolysaccharide-dependent proinflammatory Toll-like receptor 4/NLRP3 inflammasome pathway (Mastrocola et al., 2018). However, previous studies found that *Oscillospira* decreased in patients with DILI compared to those at risk of nonalcoholic fatty liver disease and controls (Rodriguez-Diaz et al., 2022). Our study supported the evidence that *Oscillospira* exerts a positive effect on DILI. It has been found that 5,6-dihydrothymine is associated with DNA damage and repair (Park et al., 2011), whereas there is no evidence of an association between 5,6-dihydrothymine and liver disease. Our study revealed the underlying mechanism that *Oscillospira* exerted a positive effect on DILI via modulating plasma 5,6-dihydrothymine levels. Besides, although salicylate was reported as a hepatotoxic drug (Doi and Horie, 2010), updated evidence showed the preventative effect of sodium salicylate nanoparticles on cisplatin-mediated hepatotoxicity (Alkhalaf et al., 2023). Furthermore, patients with DILI present with serious mitochondrial damage, resulting in the dysfunction of citric acid (Sanyal et al., 2001; Chella Krishnan et al., 2018; Fromenty, 2020). Our study showed that the salicylate-to-citrate ratio protected the liver from hepatoxicity via the above mechanisms, whereas *Oscillospira* presented a positive effect on DILI by weakening the protective effect of salicylate on the citrate ratio.

*Roseburia* exerted a positive effect on DILI. A previous study found that *Roseburia* increased in mice with d-galactosamine/lipopolysaccharide-induced acute liver injury (Liu et al., 2022), which aligned with our result. However, *Roseburia* was reported to decrease in mice with carbon tetrachloride-induced liver injury (Yang et al., 2021), though this difference could be attributed to the differences between mouse models and human patients. Moreover, we found no mediating metabolites in the effect of *Roseburia* on DILI, indicating that *Roseburia* could directly affect hepatoxicity.

Our study provided potential biomarkers for risk stratification and elucidated the underlying mechanisms for further investigation of DILI. DILI is the most common cause of acute liver failure. The outcome would be fatal for these patients without liver transplantation (European Association for the Study of the Liver et al., 2019). Moreover, ~20% of DILI patients would progress into chronicity, which would result in cirrhosis, liver failure, death, and/or liver transplantation (Lo Re et al., 2015). However, owing to the limitations of existing predictive models in terms of predictive effects and clinical feasibility, accurately predicting chronic and fatal DILI remains difficult. Our study identified the causal associations between the gut microbiota, blood metabolites, and DILI, which could serve as potential biomarkers to predict the outcomes of DILI patients. Furthermore, the mechanism of DILI is involved in hepatic metabolism and excretion of the DILI-causative agent, leading to cellular stress, cell death, activation of an adaptive immune response, and a failure to adapt (Andrade et al., 2019). Our study revealed that the gut microbiota could affect DILI via plasma metabolites, which could be the underlying mechanism for further investigation of DILI.

There are several strengths in our study. First, we used Mendelian randomization analysis to explore the associations between the gut microbiota, plasma metabolome, and toxic liver injury, which could mitigate bias from confounding factors and address issues related to reverse causality (Emdin et al., 2017). Second, we revealed that the gut microbiota could affect DILI via plasma metabolites, which could serve as potential biomarkers for risk stratification and as underlying mechanisms for further investigation of DILI. Third, we assessed causal associations in the European population to avoid bias arising from population structure.

Limitations need to be considered when interpreting our results. First, we used less stringent criteria (1 × 10^−5^) to identify instrument variables for partial exposures. However, it was reassuring that F-statistics exceeded 10, indicating that the weak instrument bias should be minimal (Pierce and Burgess, 2013). Second, the study populations were limited to those with European ancestry. Therefore, our results should be cautiously interpreted across non-European populations.

## Conclusion

We found causal associations between the gut microbiota, blood metabolites, and DILI, which could serve as potential biomarkers to predict the outcomes of DILI patients. Furthermore, we revealed that the gut microbiota could affect DILI via plasma metabolites, which could be an underlying mechanism of DILI worthy of further investigation.

## Data availability statement

The original contributions presented in the study are included in the article/Supplementary material, further inquiries can be directed to the corresponding author.

## Ethics statement

This is a secondary analysis based on summary statistics from existing, published studies. The ethical approval and informed consent have been obtained by all original studies. The studies were conducted in accordance with the local legislation and institutional requirements. The participants provided their written informed consent to participate in this study.

## Author contributions

HF: Conceptualization, Formal analysis, Investigation, Methodology, Writing – original draft. SZ: Methodology, Writing – review & editing. SS: Methodology, Writing – review & editing, Formal analysis, Investigation. QX: Writing – review & editing, Conceptualization.
