# Supplementary material for: Gut microbiota causally affects drug-induced liver injury via plasma metabolites: a Mendelian randomization study
Source: Front Microbiol. 2024 Jul 18;15:1432049. doi: 10.3389/fmicb.2024.1432049 (PMC11291454; doi:10.3389/fmicb.2024.1432049)
Supplement: Supplementary file 1 [file Data_Sheet_1.ZIP › Code and code comment/Code comment.docx]

Code 1：MR analysis of the effects of gut microbiota on DILI.

The code has been fully commented in the file. Specially, the “exposure.F.csv” refers to the information of instrument variables for gut microbiota (Supplementary table S2). The “finngen_R9_K11_TOXLIV.gz” refers to the file of DILI, which could be found in FinnGen databases (PMID: 36653562).

Code 2：MR analysis of the effects of DILI on gut microbiota.

The code has been fully commented in the file. Specially, the “exposure_DILI.csv” refers to the information of instrument variables for DILI (Supplementary table S4). The “ebi-a-GCST90016972” refers to the GWAS ID of genus Blautia, GWAS ID of other gut microbiota could be found in corresponding article (PMID: 33462485) and IEU databases (https://gwas.mrcieu.ac.uk/).

Code 3：MR analysis of the effects of blood metabolites on DILI.

The code has been fully commented in the file. Specially, the “exposure.F.csv” refers to the information of instrument variables for blood metabolites (Supplementary table S3). The “finngen_R9_K11_TOXLIV.gz” refers to the file of DILI, which could be found in FinnGen databases (PMID: 36653562).

Code 4：MR analysis of the effects of gut microbiota on blood metabolites.

The code has been fully commented in the file. Specially, the “exposure.F.csv” refers to the information of instrument variables for gut microbiota (Supplementary table S2). The “GCST90199762_buildGRCh38.tsv.gz” refers to the file of blood metabolites, which could be found in corresponding article (PMID: 36635386). The files of other blood metabolites could be found in corresponding article (PMID: 36635386).
