# Supplementary material for: Gut microbiota causally affects drug-induced liver injury via plasma metabolites: a Mendelian randomization study
Source: Front Microbiol. 2024 Jul 18;15:1432049. doi: 10.3389/fmicb.2024.1432049 (PMC11291454; doi:10.3389/fmicb.2024.1432049)
Supplement: Supplementary file 2 [file Table_1.DOCX]

**SUPPLEMENTARY MATERIALS**

**STROBE-MR checklist of recommended items to address in reports of Mendelian randomization studies**^1^ ^2^

| **Item No.** | **Section** | **Checklist item** | **Page No.** | **Relevant text from manuscript** |
| --- | --- | --- | --- | --- |
| 1 | **TITLE and ABSTRACT** | Indicate Mendelian randomization (MR) as the study’s design in the title and/or the abstract if that is a main purpose of the study | - | Gut microbiota causally affects Drug-induced liver injury via plasma metabolites: A Mendelian Randomization study |
|  | **INTRODUCTION** |  |  |  |
| 2 | **Background** | Explain the scientific background and rationale for the reported study. What is the exposure? Is a potential causal relationship between exposure and outcome plausible? Justify why MR is a helpful method to address the study question | - | In the introduction, we introduce the exposures of interest ang the rationale for using MR to explore the causal question. |
| 3 | **Objectives** | State specific objectives clearly, including pre-specified causal hypotheses (if any). State that MR is a method that, under specific assumptions, intends to estimate causal effects | - | Therefore, we performed a MR analysis to investigate investigated the causal associations between gut microbiota, plasma metabolome and toxic liver disease. |
|  | **METHODS** |  |  |  |
| 4 | **Study design and data sources** | Present key elements of the study design early in the article. Consider including a table listing sources of data for all phases of the study. For each data source contributing to the analysis, describe the following: |  |  |
|  | a) | Setting: Describe the study design and the underlying population, if possible. Describe the setting, locations, and relevant dates, including periods of recruitment, exposure, follow-up, and data collection, when available. | - | Figure 1 and Table 1 |
|  | b) | Participants: Give the eligibility criteria, and the sources and methods of selection of participants. Report the sample size, and whether any power or sample size calculations were carried out prior to the main analysis | - | Table 1 |
|  | c) | Describe measurement, quality control and selection of genetic variants | - | Table 1 |
|  | d) | For each exposure, outcome, and other relevant variables, describe methods of assessment and diagnostic criteria for diseases | - | Table 1 |
|  | e) | Provide details of ethics committee approval and participant informed consent, if relevant | - | All procedures followed were in accordance with the ethical standards of the responsible committee on human experimentation (institutional and national) and with the Helsinki Declaration of 1975, as revised in 2008. All studies included in cited genome-wide association studies had approved by a relevant review board. All participants had provided the inform consent. |
| 5 | **Assumptions** | Explicitly state the three core IV assumptions for the main analysis (relevance, independence and exclusion restriction) as well assumptions for any additional or sensitivity analysis | - | Figure 1. |
| 6 | **Statistical methods: main analysis** | Describe statistical methods and statistics used |  |  |
|  | a) | Describe how quantitative variables were handled in the analyses (i.e., scale, units, model) | - | Table 1 |
|  | b) | Describe how genetic variants were handled in the analyses and, if applicable, how their weights were selected | - | SNPs for gut microbiota and plasma metabolites were selected as instrument variables with a P-value (<5×10-5). All SNPs were clumped for independent inheritance (R2<0.001, within 10 Mb). F-statistics were calculated to asses SNP’s validity, with a threshold of exceeding 10[12]. |
|  | c) | Describe the MR estimator (e.g. two-stage least squares, Wald ratio) and related statistics. Detail the included covariates and, in case of two-sample MR, whether the same covariate set was used for adjustment in the two samples | - | GWAS data of gut microbiota were obtained from MiBioGen consortium based on 18,340 participants, of which 78% were Europeans[9]. MiBioGen consortium analyzed the genome information and 16S fecal microbiome, which resulted in 211 taxa (131 genera, 35 families, 20 orders, 16 classes and 9 phyla). And only data of 131 genera were used in our study. Moreover, GWAS data of plasma metabolome were obtained from 8,299 European individuals in Canadian Longitudinal Study on Aging cohort, including 1,091 metabolites and 309 metabolite ratios[10]. Furthermore, GWAS data of toxic liver disease were obtained from FinnGen consortium including 388 cases and 366,450 controls[11], which were diagnosed based on the International Classification of Diseases (ICD10: K71). Detailed information of corresponding data refers to the cited papers. Overlapping individuals have negligible effects on the power when selected instrument variables are strong enough[12]. |
|  | d) | Explain how missing data were addressed |  | - |
|  | e) | If applicable, indicate how multiple testing was addressed |  | - |
| 7 | **Assessment of assumptions** | Describe any methods or prior knowledge used to assess the assumptions or justify their validity | - | F-statistics were calculated to asses SNP’s validity, with a threshold of exceeding 10[12]. |
| 8 | **Sensitivity analyses and additional analyses** | Describe any sensitivity analyses or additional analyses performed (e.g. comparison of effect estimates from different approaches, independent replication, bias analytic techniques, validation of instruments, simulations) | - | Univariable Mendelian Randomization analysis was used to explore the causal effects of gut microbiota and plasma metabolome on toxic liver diseases. Five methods [Inverse-variance-weighted, MR-Egger, Weighted median, Simple mode, and Weighted mode] were used to evaluate and validate causal effects, which assumptions and advantages was summarized in Table S1. Inverse-variance-weighted was the primary method. Cochran's Q statistic was preformed to evaluate heterogeneity. The MR-Egger intercept was preformed to evaluate pleiotropy. Only causal effect with Inverse-variance-weighted P-value<0.05 and without heterogeneity and pleiotropy (corresponding P-value>0.05) was identified significant effect. Moreover, based on above criteria, we selected more stringent criteria with the same direction in five methods in the analysis of associations between plasma metabolome and toxic liver disease. Furthermore, we used MetaboAnalyst 5.0 (https://www.metaboanalyst.ca/) to implement the enrichiment analysis of identified metabolites.  Two-step Mendelian Randomization[13] was used to explore the mediation roles of plasma metabolites in the causal effects of gut microbiota on toxic liver disease. Based on causal effects of gut microbiota on toxic liver disease, two causal effects were explored: the causal effects of mediators (plasma metabolites) on toxic liver disease and causal effects of exposures (gut microbiota) on mediators. Where there was evidence that gut microbiota affected plasma metabolites, which in turn affect the toxic liver disease, we used product of coefficient method to evaluate mediation effects. Standard errors for mediation effects were derived using delta method.  All tests were two-sided and performed using the TwoSampleMR (version 0.5.7), packages in the R software (version 4.0.2). A P value <0.05 indicates statistical significance. |
| 9 | **Software and pre-registration** |  |  |  |
|  | a) | Name statistical software and package(s), including version and settings used | - | All tests were two-sided and performed using the TwoSampleMR (version 0.5.7), packages in the R software (version 4.0.2). A P value <0.05 indicates statistical significance. |
|  | b) | State whether the study protocol and details were pre-registered (as well as when and where) | - | This is a secondary analysis based on summary statistics from existing, published studies. The ethical approval and informed consent have been obtained by all original studies |
|  | **RESULTS** |  |  |  |
| 10 | **Descriptive data** |  |  |  |
|  | a) | Report the numbers of individuals at each stage of included studies and reasons for exclusion. Consider use of a flow diagram | - | Table 1 |
|  | b) | Report summary statistics for phenotypic exposure(s), outcome(s), and other relevant variables (e.g. means, SDs, proportions) | - | Table 1 |
|  | c) | If the data sources include meta-analyses of previous studies, provide the assessments of heterogeneity across these studies | - | Table 1 |
|  | d) | For two-sample MR:  i.  Provide justification of the similarity of the genetic variant-exposure associations between the exposure and outcome samples  ii.  Provide information on the number of individuals who overlap between the exposure and outcome studies | -  - | i. Third, we assessed causal connections in the European population to avoid bias arising from population structure.  ii. Overlapping individuals have negligible effects on the power when selected instrument variables are strong enough[12]. |
| 11 | **Main results** |  |  |  |
|  | a) | Report the associations between genetic variant and exposure, and between genetic variant and outcome, preferably on an interpretable scale | - | Figure2-4, Table 2, and Table S2 |
|  | b) | Report MR estimates of the relationship between exposure and outcome, and the measures of uncertainty from the MR analysis, on an interpretable scale, such as odds ratio or relative risk per SD difference | - | Most of the resulting space is a report on the contents of the article. |
|  | c) | If relevant, consider translating estimates of relative risk into absolute risk for a meaningful time period | - | - |
|  | d) | Consider plots to visualize results (e.g. forest plot, scatterplot of associations between genetic variants and outcome versus between genetic variants and exposure) | - | Main results are presented in figures. |
| 12 | **Assessment of assumptions** |  |  |  |
|  | a) | Report the assessment of the validity of the assumptions | - | Univariable Mendelian Randomization analysis was used to explore the causal effects of gut microbiota and plasma metabolome on toxic liver diseases. Five methods [Inverse-variance-weighted, MR-Egger, Weighted median, Simple mode, and Weighted mode] were used to evaluate and validate causal effects, which assumptions and advantages was summarized in Table S1. Inverse-variance-weighted was the primary method. Cochran's Q statistic was preformed to evaluate heterogeneity. The MR-Egger intercept was preformed to evaluate pleiotropy. Only causal effect with Inverse-variance-weighted P-value<0.05 and without heterogeneity and pleiotropy (corresponding P-value>0.05) was identified significant effect. Moreover, based on above criteria, we selected more stringent criteria with the same direction in five methods in the analysis of associations between plasma metabolome and toxic liver disease. Furthermore, we used MetaboAnalyst 5.0 (https://www.metaboanalyst.ca/) to implement the enrichiment analysis of identified metabolites.  Two-step Mendelian Randomization[13] was used to explore the mediation roles of plasma metabolites in the causal effects of gut microbiota on toxic liver disease. Based on causal effects of gut microbiota on toxic liver disease, two causal effects were explored: the causal effects of mediators (plasma metabolites) on toxic liver disease and causal effects of exposures (gut microbiota) on mediators. Where there was evidence that gut microbiota affected plasma metabolites, which in turn affect the toxic liver disease, we used product of coefficient method to evaluate mediation effects. Standard errors for mediation effects were derived using delta method.  All tests were two-sided and performed using the TwoSampleMR (version 0.5.7), packages in the R software (version 4.0.2). A P value <0.05 indicates statistical significance. |
|  | b) | Report any additional statistics (e.g., assessments of heterogeneity across genetic variants, such as *I^2^*, Q statistic or E-value) | - | See above |
| 13 | **Sensitivity analyses and additional analyses** |  |  |  |
|  | a) | Report any sensitivity analyses to assess the robustness of the main results to violations of the assumptions | - | See above |
|  | b) | Report results from other sensitivity analyses or additional analyses | - | See above |
|  | c) | Report any assessment of direction of causal relationship (e.g., bidirectional MR) | - | - |
|  | d) | When relevant, report and compare with estimates from non-MR analyses | - | We have described these contents in Discussion. |
|  | e) | Consider additional plots to visualize results (e.g., leave-one-out analyses) | - | - |
|  | **DISCUSSION** |  |  |  |
| 14 | **Key results** | Summarize key results with reference to study objectives | - | In this study, we systemically investigated the causal effects of gut microbiota and blood metabolites on toxic liver disease. We revealed that 5 taxa and 53 metabolites were causally associated with toxic liver disease. Further analysis showed that 4 taxa affected toxic liver disease via 5 mediation metabolites, which could serve as potential biomarkers for risk stratification and underlying mechanism for further investigation of toxic liver disease. |
| 15 | **Limitations** | Discuss limitations of the study, taking into account the validity of the IV assumptions, other sources of potential bias, and imprecision. Discuss both direction and magnitude of any potential bias and any efforts to address them | - | Limitations need consideration when interpreting our results. First, we used less stringent criteria (5×10-5) to identify instrument variables for partial exposures. However, it was reassuring that F-statistics exceed 10, indicating the weak instrument bias should be minimal[12]. Second, the correct for multiple testing was not performed in this exploratory study. Third, study population of our study were limited to European ancestry. Therefore, our results should be cautiously interpreted across non-European population. |
| 16 | **Interpretation** |  |  |  |
|  | a) | Meaning: Give a cautious overall interpretation of results in the context of their limitations and in comparison with other studies | - | We revealed that gut microbiota could affect toxic liver disease via plasma metabolites, which could serve as potential biomarkers for risk stratification and underlying mechanism for further investigation of toxic liver disease. |
|  | b) | Mechanism: Discuss underlying biological mechanisms that could drive a potential causal relationship between the investigated exposure and the outcome, and whether the gene-environment equivalence assumption is reasonable. Use causal language carefully, clarifying that IV estimates may provide causal effects only under certain assumptions | - | We have described these contents in Discussion. |
|  | c) | Clinical relevance: Discuss whether the results have clinical or public policy relevance, and to what extent they inform effect sizes of possible interventions | - | We revealed that gut microbiota could affect toxic liver disease via plasma metabolites, which could serve as potential biomarkers for risk stratification and underlying mechanism for further investigation of toxic liver disease. |
| 17 | **Generalizability** | Discuss the generalizability of the study results (a) to other populations, (b) across other exposure periods/timings, and (c) across other levels of exposure | - | - |
|  | **OTHER INFORMATION** |  |  |  |
| 18 | **Funding** | Describe sources of funding and the role of funders in the present study and, if applicable, sources of funding for the databases and original study or studies on which the present study is based | - | We have described these contents in Funding. |
| 19 | **Data and data sharing** | Provide the data used to perform all analyses or report where and how the data can be accessed, and reference these sources in the article. Provide the statistical code needed to reproduce the results in the article, or report whether the code is publicly accessible and if so, where | - | We have described these contents in Availability of data and materials. |
| 20 | **Conflicts of Interest** | All authors should declare all potential conflicts of interest | - | The authors declare that they have no competing interests. |

This checklist is copyrighted by the Equator Network under the Creative Commons Attribution 3.0 Unported (CC BY 3.0) license.

1. Skrivankova VW, Richmond RC, Woolf BAR, Yarmolinsky J, Davies NM, Swanson SA, et al. Strengthening the Reporting of Observational Studies in Epidemiology using Mendelian Randomization (STROBE-MR) Statement. JAMA. 2021;under review.

2. Skrivankova VW, Richmond RC, Woolf BAR, Davies NM, Swanson SA, VanderWeele TJ, et al. Strengthening the Reporting of Observational Studies in Epidemiology using Mendelian Randomisation (STROBE-MR): Explanation and Elaboration. BMJ. 2021;375:n2233.
